# Continuous glucose monitoring with Humalog Mix 25 versus Humalog Mix 50, twice daily: A comparative pilot study -Results from the Jikei-EValuation of insulin Lispro mixture on pharmacodynamics and glycemic VariancE (J-EVOLVE) study

**DOI:** 10.1186/1475-2840-9-16

**Published:** 2010-05-03

**Authors:** Rimei Nishimura, Daisuke Tsujino, Kentaro Taki, Aya Morimoto, Naoko Tajima

**Affiliations:** 1 Division of Diabetes, Metabolism and Endocrinology, Department of Internal Medicine, Jikei University School of Medicine, Tokyo, Japan

## Abstract

**Objective:**

To evaluate glycemic variability associated with two different premixed insulin analogue formulations when used in a twice-daily regimen.

**Patients and Methods:**

Subjects comprised type 2 diabetic patients aged 20-79 years, treated with twice daily premixed insulin or insulin analogue formulations. All subjects were hospitalized for 6 days and randomized to receive either Humalog Mix 25 (Mix 25) or Humalog Mix 50 (Mix 50). They were then crossed over to the other arm between day 3 and day 4 of the study. Continuous glucose monitoring (CGM) was performed on all subjects to examine the differences in glycemic variability.

**Results:**

Eleven type 2 diabetic patients were enrolled. No significant difference was found in 24-hour mean glucose values and their SDs, pre-meal glucose values, increases from pre-meal to peak glucose values, or time to peak glucose levels between either group. However, the mean glucose values observed during 0-8 hrs were significantly lower with Mix 25 compared to Mix 50 (128 vs. 147 mg/dL; p = 0.024).

**Conclusions:**

The twice-daily Mix 25 regimen provided superior overnight glycemic control compared to the Mix 50 regimen in Japanese patients with type 2 diabetes. However, both twice-daily regimens with either Mix 25 or Mix 50 provided inadequate post-lunch glycemic control.

**Trial Registration:**

Current Controlled Trials UMIN000001327

## Introduction

Results from the Diabetes Control Complications Trial (DCCT) [1] and the Kumamoto Study [2] demonstrated that in both type 1 and type 2 diabetes, intensive insulin therapy combining regular- and intermediate-acting insulin formulations, provides a significantly greater improvement in HbA1c values and reduces the onset and progression of diabetes-associated microangiopathy to a greater degree, than conventional insulin therapy. These findings have led to intensive insulin therapy being proactively and increasingly used in diabetic patients.

Following these results, several clinical studies involving insulin analogue mixtures have been conducted, which have consistently demonstrated that there is no significant difference in efficacy between twice-daily regimens with insulin analogue mixtures and intensive insulin therapy. Representative of these, the 4T Study [3] showed no significant difference in improvement in HbA1c values between patients treated with intensive insulin therapy plus an oral anti-diabetic drug (OADD), and those treated with a twice-daily mixed insulin analogue regimen plus an OADD. Similar results have been reported from two independent Japanese studies, the JOINT and JDDM11 Studies [4,5], conducted in patients with sulfonylurea secondary failure, which compared the efficacy between intensive insulin therapy and twice-daily regimens with insulin analogue mixtures. These results suggested that twice-daily regimens with insulin analogue mixtures are as effective as intensive insulin therapy in improving HbA1c values.

In recent years, insulin analogues combined at different blend ratios (Humalog Mix 25: 25% lispro, 75% protaminated lispro, "Mix 25" hereafter; Humalog Mix 50: 50% lispro, 50% protaminated lispro, "Mix 50" hereafter) have become commercially available. However there have been no reports comparing the effect of these premixed insulin analogue formulations on glycemic variability when used in a twice-daily regimen. Therefore, we conducted a cross-over study in type 2 diabetic subjects treated with an identical diet, and used continuous glucose monitoring (CGM) to compare glycemic variability associated with a twice-daily regimen of either Mix 25 or. Mix 50.

## Patients and Methods

**Figure 1:**
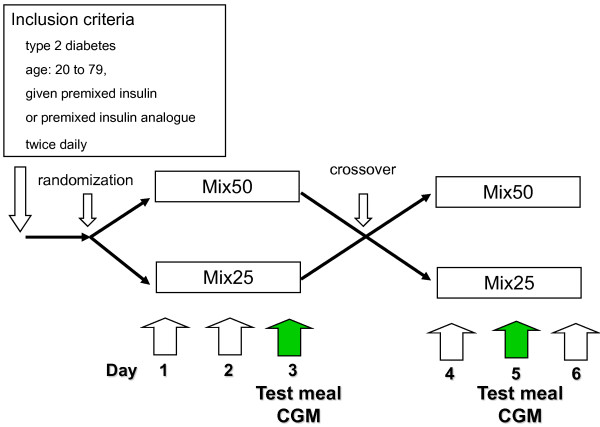
**Study protocol**.

Study subjects comprised type 2 diabetic patients aged between 20 and less than 80 years old, who were treated with premixed insulin preparations or premixed insulin analogue formulations, twice daily. Informed consent was obtained from all patients prior to starting the study. Subjects were hospitalized during the 6-day study period, and were randomized to receive either Mix 25 or Mix 50. On Day 3-4 subjects crossed over to the other study arm. Insulin injections were given twice daily 0-15 min before breakfast and before supper. All subjects received the same insulin dose during hospitalization (Figure 1).

The subjects received test meals consisting of the same nutrients and equivalent caloric intake on days 3 and 5. The total caloric intake for subjects was defined by the attending physicians, as 1,440 kcal (group 1), 1600 kcal (group 2), or 1,840 kcal (group 3) per day to accommodate differences in physique among the subjects. A standardized test meal was given for breakfast, irrespective of differences in physique (total calories, 460 kcal; carbohydrates, 49.1%; proteins, 15.7%; and lipids, 35.2%) [6] and commonly available foods were given for lunch and supper.

Midday meals accounted for a total caloric intake of 455 kcal (carbohydrates, 62.2%; proteins, 16.6%; and lipids, 21.2%) in group 1; 571 kcal (carbohydrates, 59.9%; proteins, 15.7%; and lipids, 24.4%) in group 2; and 684 kcal (carbohydrates, 65.2%; proteins, 14.1%; and lipids, 20.7%) in group 3.

Evening meals accounted for a total caloric intake of 465 kcal (carbohydrates, 56.9%; proteins, 21.6%; and lipids, 21.5%) in group 1; 589 kcal (carbohydrates, 57.7%; proteins, 20.3%; and lipids, 22.0%) in group 2; and 702 kcal (carbohydrates, 63.2%; proteins, 14.1%; and lipids, 18.7%) in group 3.

The subjects were instructed to refrain from excessive physical activity, and to maintain a similar level of physical activity on days 3 and 5 when the test meals were given.

CGM was performed on all subjects using the CGMS system GOLD (Medtronic Inc.) [7], which measures blood glucose every 10 seconds, recording mean values every 5 minutes. Therefore, 288 measurements were recorded daily, providing sufficient information to understand diurnal blood glucose variations. The mean glucose concentrations and their standard deviations (SDs) were calculated using the CGMS data from 0-24 hrs on day 3 and day 5. Mean 24-hour glucose concentrations and their SDs, pre-meal glucose concentrations, postprandial peak glucose concentrations, increases from pre-meal to peak glucose concentrations, and the time from pre-meal to peak glucose concentrations were compared using the Student t-test. Additionally, mean glucose values were compared between overnight values (from 0-8 hrs) and those obtained from 8-24 hrs. At the end of the study, CGM results were disclosed to all subjects, and the insulin regimen was chosen for each patient on the basis of his/her preference. Statistical analyses were performed using SPSS 16.0 [8]. The current study was approved by the Jikei University School of Medicine Ethics Committee and was registered as Clinical Trial UMIN000001327 [9].

## Results

Subjects comprised 11 type 2 diabetic patients (males: females, 9:2; mean age, 63.9 ± 7.8 years; mean BMI, 24.0 ± 4.0; mean duration of diabetes, 18.0 ± 12.0 years; mean U-CPR, 44.3 ± 37.8 μg/day; mean baseline HbA1c value, 8.4 ± 2.1%; and mean total daily insulin dose, 24.9 ± 8.9 units) (Table 1).

**Table 1:** Baseline characteristics of the study subjects and their total daily insulin doses

Subjects (n)	11
Male (%)	9 (81.9)
HbA1c value (%) at CGM	8.4 ± 2.1
Age (years-old)	63.9 ± 7.8
Body mass index (kg/m^2^)	24.0 ± 4.0
Duration of diabetes (years)	18.0 ± 12.0
Urinary C-peptide (μg/day)	44.3 ± 37.8
Total insulin dose (U/day)	24.9 ± 8.9
Morning insulin dose (U)	13.7 ± 5.2
Evening insulin dose (U)	11.1 ± 4.0
Morning/evening insulin dose ratio	1.24 ± 0.27

Values are represented as mean ± SD or ratio (%).

No significant difference was found in 24-hour mean glucose concentrations and their SDs between those given Mix 25 and those given Mix 50 (176 vs. 187 mg/dL; 50 vs. 49 mg/dL). Similarly, there was no significant difference between Mix 25 and Mix 50 in pre-meal glucose concentrations (144 vs. 172 mg/dL in the morning, 151 vs. 153 mg/dL in the daytime, and 161 vs. 177 mg/dL in the evening); increase from pre-meal to peak glucose concentrations (93 vs. 85 mg/dL in the morning, 113 vs. 138 mg/dL in the daytime, and 76 vs. 59 mg/dL in the evening); and time to peak glucose concentrations (98 vs. 93 minutes in the morning, 136 vs. 131 minutes in the daytime, and 81 vs. 71 minutes in the evening) (Table 2).

**Table 2:** Indices (mean ± SD) for glycemic variation with twice-daily Mix 25 vs. Mix 50 regimens in 11 type 2 diabetic patients

	Mix 25 regimen	Mix 50 regimen	*P *value*
24-hour mean glucose concentration (mg/dL)	176 ± 54	187 ± 41	0.231
SD for 24-hour mean glucose concentration (mg/dL)	50 ± 16	49 ± 11	0.962
Mean glucose concentration 0-8 hrs (mg/dL)	127 ± 52	147 ± 42	0.024
Mean glucose concentration 8-24 hrs (mg/dL)	199 ± 59	206 ± 45	0.514
Lowest glucose concentration (mg/dL)	95 ± 49	105 ± 29	0.464
Peak glucose concentration (mg/dL)	276 ± 62	299 ± 43	0.061

Mean pre-meal glucose concentration (mg/dL)			
Pre-breakfast	144 ± 54	172 ± 45	0.496
Pre-lunch	151 ± 72	153 ± 55	0.349
Pre-supper	161 ± 60	177 ± 60	0.997

Increase in postprandial peak glucose concentration (mg/dL)			
Post-breakfast	93 ± 35	85 ± 28	0.366
Post-lunch	113 ± 43	138 ± 46	0.949
Post-supper	76 ± 28	59 ± 36	0.287

Time to postprandial peak glucose concentration (minutes)			
Post-breakfast	98 ± 38	93 ± 37	0.667
Post-lunch	136 ± 34	131 ± 21	0.658
Post-supper	81 ± 23	71 ± 21	0.358

*t-test

**Figure 2:**
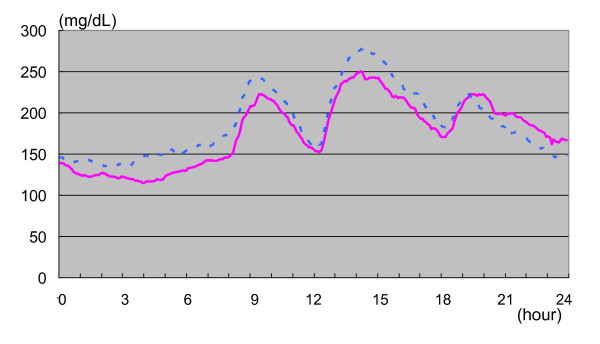
**Changes in mean glucose concentrations over time with twice-daily Mix 25 (solid line) vs. Mix 50 (dotted line) regimens in 11 type 2 diabetic patients**.

However, mean glucose concentrations were significantly lower with Mix 25 compared to Mix 50 (128 vs. 147 mg/dL; *P *= 0.024) in the overnight subgroup analysis compared to those for the 8-24 hr period. When changes in the 24-hour mean glucose value were plotted over time, the overnight glucose level was more favorable with Mix 25 (Figure 2). However, despite this, asymptomatic overnight hypoglycemia 60 mg/dL or lower was observed in 2 patients (18%) with Mix 25, while no overnight hypoglycemia was observed with Mix 50.

At the end of the study, when patients were shown their CGM data and asked to choose their insulin regimen, more patients (7 patients; 63.6%) chose Mix 25 over Mix 50, given that better glycemic control was noted with Mix 25 overnight while it was not significantly different from that with Mix 50 during the day.

## Discussion

A detailed analysis of differences in glycemic variability with Mix 25 versus Mix 50 was performed using CGM in type 2 diabetic patients on insulin therapy.

The mean overnight glucose concentration was significantly higher with Mix 50 than Mix 25, with the pre-breakfast glucose value tending to be higher with Mix 50. However, the increases in glucose after breakfast were non-significantly smaller with Mix 50, which had a greater rapid insulin component than Mix 25. The pre-lunch glucose values were similar for both groups.

In contrast, the increase in glucose concentration after lunch was smaller with Mix 25, which had a greater intermediate-acting insulin component. This resulted in the pre-supper glucose values tending to be slightly higher with Mix 50.

The increases in the evening glucose concentration were smaller with Mix 50, similar to that noted after breakfast, although these increases were not significantly different from those reported for Mix 25.

Study results clearly demonstrated that the changes in overnight glucose concentration were significantly lower with Mix 25, which had a greater intermediate-acting insulin component than Mix 50. The differences in overnight changes between Mix 25 and Mix 50 were evident in each study participant, which led to a non-significantly greater proportion of the study participants (63.6%) choosing Mix 25 over Mix 50. However, patients need to be aware that Mix 25 is associated with a potentially greater risk for asymptomatic overnight hypoglycemia.

Post-prandial increases in glucose concentration reflected the differing blend ratios of rapid insulin between Mix 25 and Mix 50, although this did not result in significant differences. Interestingly, however, the time to peak glucose concentration did not clearly reflect these differing rapid insulin blend ratios, suggesting that these ratios mainly affected the post-meal increases in glucose concentration.

Our study results also demonstrated that twice-daily Mix 25 or Mix 50 regimens provided inadequate glycemic control after lunch for approximately 4 hours. This suggests that insulin analogue injections 3 times daily are preferable to 2 times daily to achieve improved glycemic control and furthermore that Mix 25 injected 3 times daily or Mix 50 injected before breakfast and lunch followed by Mix 25 before supper could represent an ideal insulin regimen. In this regard, Rosenstock et al compared a 3 times-daily Mix 50 regimen to intensive insulin therapy (glargine at bedtime plus mealtime lispro) in patients with inadequate glycemic control [10]. Similar findings were observed between the two arms with regard to improvement in HbA1c values or diurnal variations in blood glucose concentration.

In regard to differences in glycemic variation resulting from differing blend ratios of mixed insulin analogue preparations, Hirao et al [11] reported on a cross-over study comparing biphasic insulin aspart 50 (BIAsp50) and 30 (BIAsp30) in 10 patients with type 2 diabetes. Using the euglycemic clamp technique, he demonstrated that both the serum insulin level and glucose infusion rate (GIR) were higher with BIAsp50 than with BIAsp30.

To the best of our knowledge, there are only two studies reported in the literature comparing premixed insulin analogue formulations and premixed insulin formulations. These were a cross-over study of biphasic insulin aspart 30 (BIAsp30) versus biphasic human insulin 30 (BHI 30) in 13 type 2 diabetic patients [12] and a randomized controlled trial of BIAsp 30 versus BHI 30 in 294 patients with type 1 and type 2 diabetes [13]. Results from both studies demonstrated that postprandial glucose increases were suppressed to a significantly greater extent with BIAsp30 than with BHI 30.

Limitations of the present study include the following: study subjects had a relatively long mean duration of disease (18 years) and it is possible that the results may be different if patients with a shorter duration of disease were included; that although a cross-over study design was used, it may not have been adequately powered given the small sample size (11 patients); and that differences detected may have been found to be significant if more patients were included.

In conclusion, our study clearly demonstrates that a twice-daily Mix 25 regimen provides superior overnight glycemic control compared to twice-daily Mix 50 in Japanese patients with type 2 diabetes. However, the twice-daily regimen of both Mix 25 and Mix 50 provided inadequate post-lunch glycemic control. A randomized comparative trial of 3 times-daily regimens with Mix 25 versus Mix 50 in Asian patients needs to be carried out in the future in order to establish insulin regimens that provide better glycemic control.

## Competing interests

The authors declare that they have no competing interests.

## Authors' contributions

RN and NT participated in the design of the study. RN performed the statistical analyses. RN, DT, KT, and AM participated in the coordination of the study. RN helped to draft the manuscript. All authors have read and approved the final manuscript.
